# Supplementary material for: Complex‐centric proteome profiling by SEC‐SWATH‐MS
Source: Mol Syst Biol. 2019 Jan 14;15(1):e8438. doi: 10.15252/msb.20188438 (PMC6346213; doi:10.15252/msb.20188438)
Supplement: Supplementary file 8 — Dataset EV7 [file MSB-15-e8438-s008.zip › feature_plots_string/Q8IWX5.pdf]

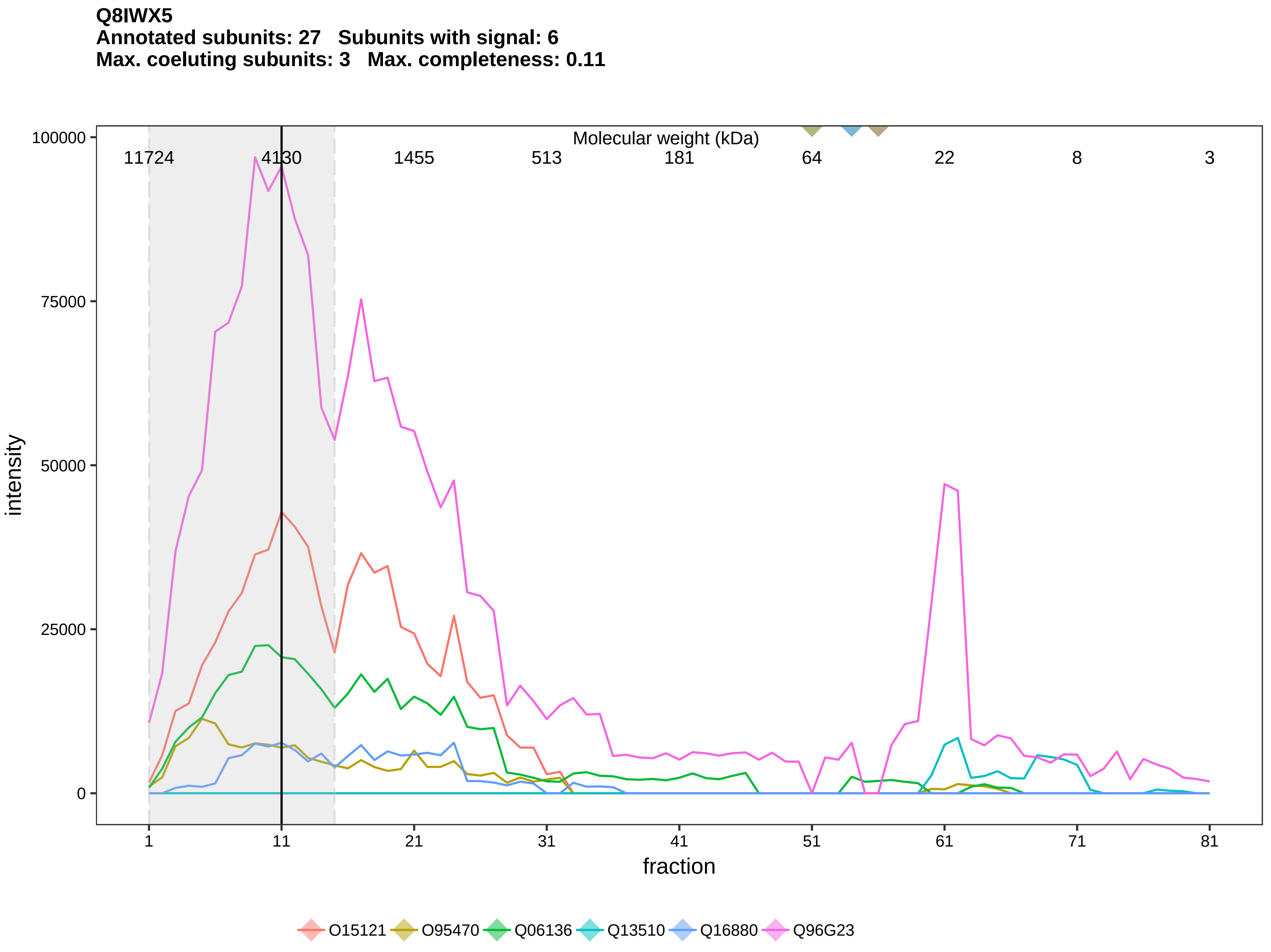

Q8IWX5
Annotated subunits: 27   Subunits with signal: 6
Max. coeluting subunits: 3   Max. completeness: 0.11
Molecular weight (kDa)
11724
4130
1455
513
181
64
22
8
3
100000
75000
50000
25000
0
intensity
1
11
21
31
41
51
61
71
81
fraction
O15121
O95470
Q06136
Q13510
Q16880
Q96G23
